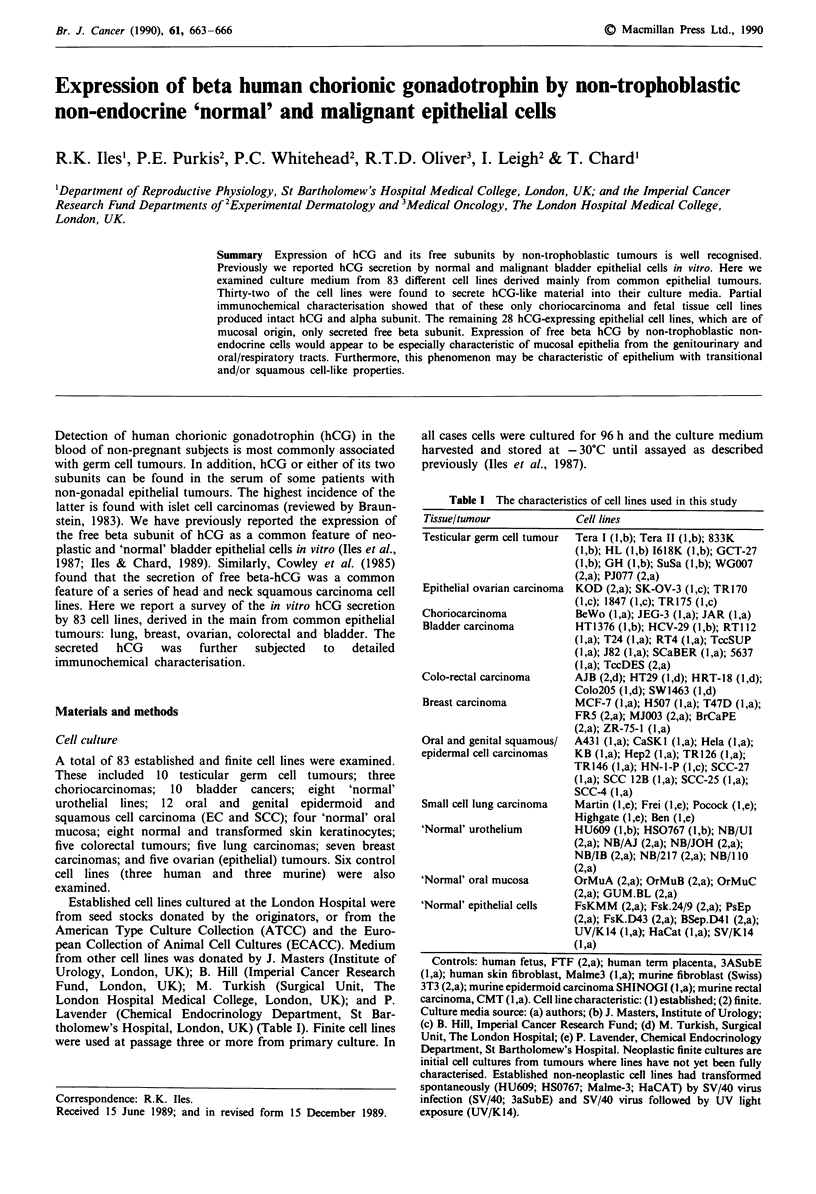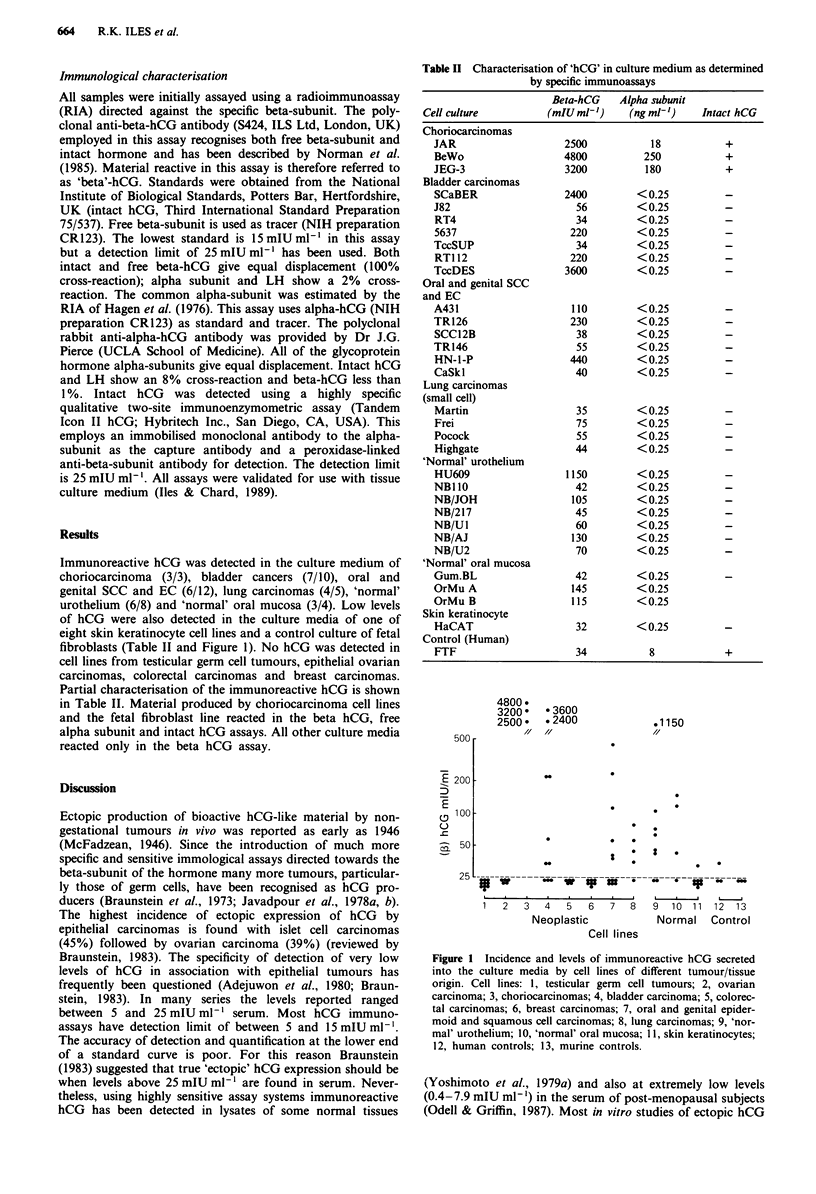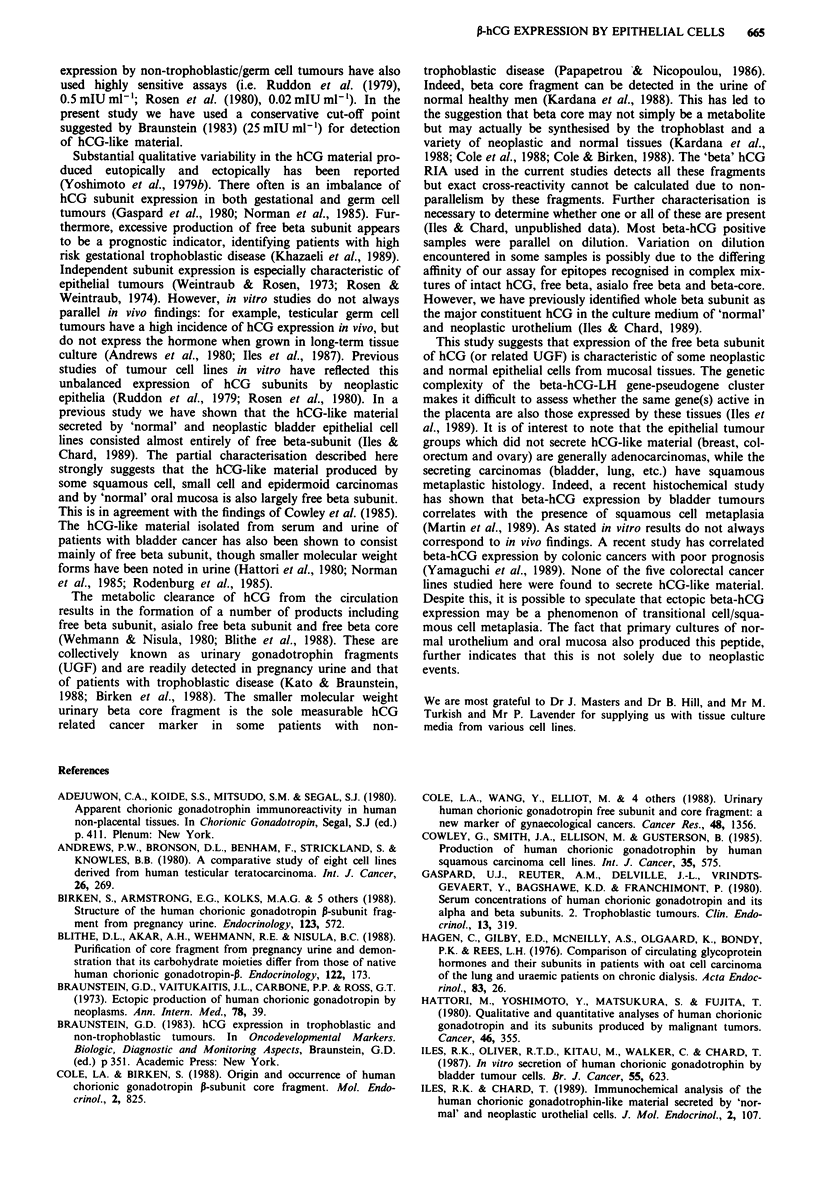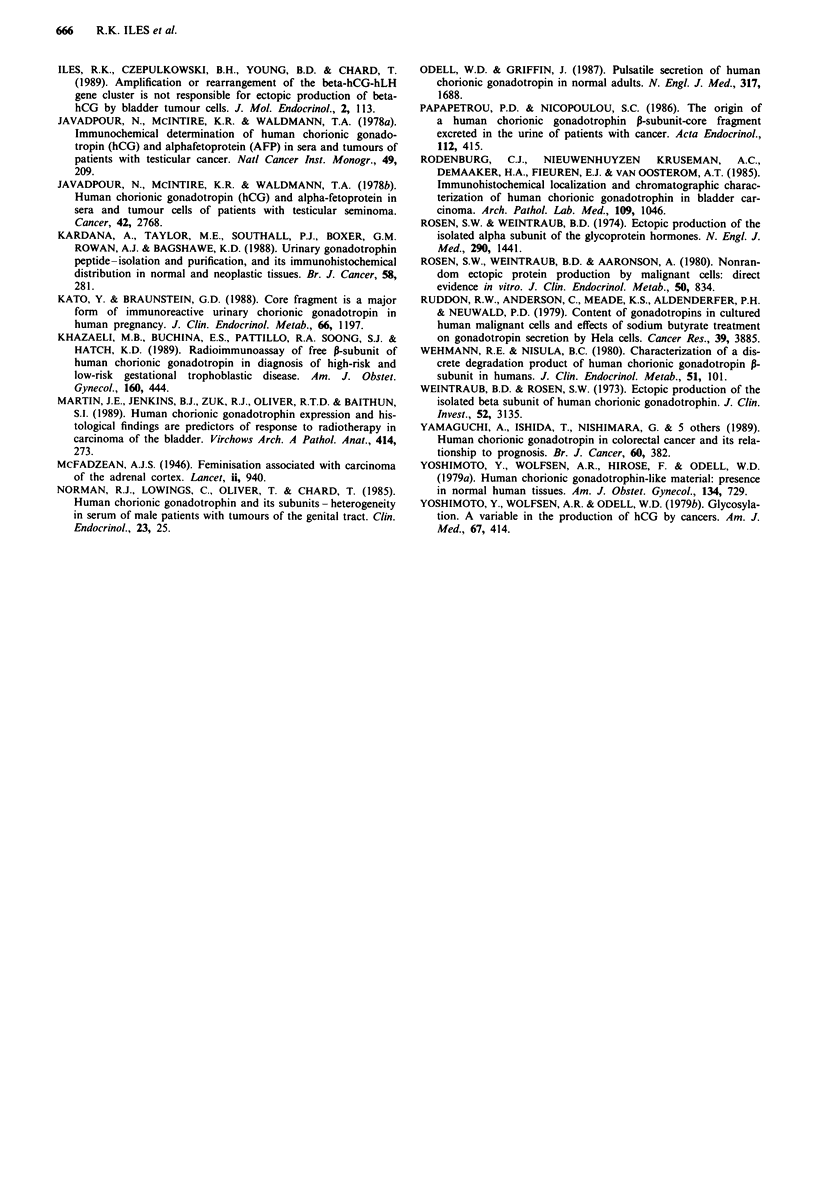# Expression of beta human chorionic gonadotrophin by non-trophoblastic non-endocrine 'normal' and malignant epithelial cells.

**DOI:** 10.1038/bjc.1990.150

**Published:** 1990-05

**Authors:** R. K. Iles, P. E. Purkis, P. C. Whitehead, R. T. Oliver, I. Leigh, T. Chard

**Affiliations:** Department of Reproductive Physiology, St Bartholomew's Hospital Medical College, London, UK.

## Abstract

Expression of hCG and its free subunits by non-trophoblastic tumours is well recognised. Previously we reported hCG secretion by normal and malignant bladder epithelial cells in vitro. Here we examined culture medium from 83 different cell lines derived mainly from common epithelial tumours. Thirty-two of the cell lines were found to secrete hCG-like material into their culture media. Partial immunochemical characterisation showed that of these only choriocarcinoma and fetal tissue cell lines produced intact hCG and alpha subunit. The remaining 28 hCG-expressing epithelial cell lines, which are of mucosal origin, only secreted free beta subunit. Expression of free beta hCG by non-trophoblastic nonendocrine cells would appear to be especially characteristic of mucosal epithelia from the genitourinary and oral/respiratory tracts. Furthermore, this phenomenon may be characteristic of epithelium with transitional and/or squamous cell-like properties.


Br. J. Cancer (1990), 61, 663-666 © Macmillan Press Ltd., 1990

### Expression of beta human chorionic gonadotrophin by non-trophoblastic non-endocrine 'normal' and malignant epithelial cells

R.K. Iles[1], P.E. Purkis[2], P.C. Whitehead[2], R.T.D. Oliver[3], I. Leigh[2] & T. Chard[1]

[1]*Department of Reproductive Physiology, St Bartholomew's Hospital Medical College, London, UK; and the Imperial Cancer Research Fund Departments of* [2]*Experimental Dermatology and* [3]*Medical Oncology, The London Hospital Medical College, London, UK.*

**Summary** Expression of hCG and its free subunits by non-trophoblastic tumours is well recognised. Previously we reported hCG secretion by normal and malignant bladder epithelial cells *in vitro*. Here we examined culture medium from 83 different cell lines derived mainly from common epithelial tumours. Thirty-two of the cell lines were found to secrete hCG-like material into their culture media. Partial immunochemical characterisation showed that of these only choriocarcinoma and fetal tissue cell lines produced intact hCG and alpha subunit. The remaining 28 hCG-expressing epithelial cell lines, which are of mucosal origin, only secreted free beta subunit. Expression of free beta hCG by non-trophoblastic non-endocrine cells would appear to be especially characteristic of mucosal epithelia from the genitourinary and oral/respiratory tracts. Furthermore, this phenomenon may be characteristic of epithelium with transitional and/or squamous cell-like properties.

Detection of human chorionic gonadotrophin (hCG) in the blood of non-pregnant subjects is most commonly associated with germ cell tumours. In addition, hCG or either of its two subunits can be found in the serum of some patients with non-gonadal epithelial tumours. The highest incidence of the latter is found with islet cell carcinomas (reviewed by Braunstein, 1983). We have previously reported the expression of the free beta subunit of hCG as a common feature of neoplastic and 'normal' bladder epithelial cells *in vitro* (Iles *et al.*, 1987; Iles & Chard, 1989). Similarly, Cowley *et al.* (1985) found that the secretion of free beta-hCG was a common feature of a series of head and neck squamous carcinoma cell lines. Here we report a survey of the *in vitro* hCG secretion by 83 cell lines, derived in the main from common epithelial tumours: lung, breast, ovarian, colorectal and bladder. The secreted hCG was further subjected to detailed immunochemical characterisation.

### Materials and methods

### *Cell culture*

A total of 83 established and finite cell lines were examined. These included 10 testicular germ cell tumours; three choriocarcinomas; 10 bladder cancers; eight 'normal' urothelial lines; 12 oral and genital epidermoid and squamous cell carcinoma (EC and SCC); four 'normal' oral mucosa; eight normal and transformed skin keratinocytes; five colorectal tumours; five lung carcinomas; seven breast carcinomas; and five ovarian (epithelial) tumours. Six control cell lines (three human and three murine) were also examined.

Established cell lines cultured at the London Hospital were from seed stocks donated by the originators, or from the American Type Culture Collection (ATCC) and the European Collection of Animal Cell Cultures (ECACC). Medium from other cell lines was donated by J. Masters (Institute of Urology, London, UK); B. Hill (Imperial Cancer Research Fund, London, UK); M. Turkish (Surgical Unit, The London Hospital Medical College, London, UK); and P. Lavender (Chemical Endocrinology Department, St Bartholomew's Hospital, London, UK) (Table I). Finite cell lines were used at passage three or more from primary culture. In all cases cells were cultured for 96 h and the culture medium harvested and stored at −30°C until assayed as described previously (Iles *et al.*, 1987).

**Table I** The characteristics of cell lines used in this study

| *Tissue/tumour* | *Cell lines* |
|---|---|
| Testicular germ cell tumour | Tera I (1,b); Tera II (1,b); 833K (1,b); HL (1,b) I618K (1,b); GCT-27 (1,b); GH (1,b); SuSa (1,b); WG007 (2,a); PJ077 (2,a) |
| Epithelial ovarian carcinoma | KOD (2,a); SK-OV-3 (1,c); TR170 (1,c); 1847 (1,c); TR175 (1,c) |
| Choriocarcinoma | BeWo (1,a); JEG-3 (1,a); JAR (1,a) |
| Bladder carcinoma | HT1376 (1,b); HCV-29 (1,b); RT112 (1,a); T24 (1,a); RT4 (1,a); TccSUP (1,a); J82 (1,a); SCaBER (1,a); 5637 (1,a); TccDES (2,a) |
| Colo-rectal carcinoma | AJB (2,d); HT29 (1,d); HRT-18 (1,d); Colo205 (1,d); SW1463 (1,d) |
| Breast carcinoma | MCF-7 (1,a); H507 (1,a); T47D (1,a); FR5 (2,a); MJ003 (2,a); BrCaPE (2,a); ZR-75-1 (1,a) |
| Oral and genital squamous/ epidermal cell carcinomas | A431 (1,a); CaSK1 (1,a); Hela (1,a); KB (1,a); Hep2 (1,a); TR126 (1,a); TR146 (1,a); HN-1-P (1,c); SCC-27 (1,a); SCC 12B (1,a); SCC-25 (1,a); SCC-4 (1,a) |
| Small cell lung carcinoma | Martin (1,e); Frei (1,e); Pocock (1,e); Highgate (1,e); Ben (1,e) |
| 'Normal' urothelium | HU609 (1,b); HSO767 (1,b); NB/UI (2,a); NB/AJ (2,a); NB/JOH (2,a); NB/IB (2,a); NB/217 (2,a); NB/110 (2,a) |
| 'Normal' oral mucosa | OrMuA (2,a); OrMuB (2,a); OrMuC (2,a); GUM.BL (2,a) |
| 'Normal' epithelial cells | FsKMM (2,a); Fsk.24/9 (2,a); PsEp (2,a); FsK.D43 (2,a); BSep.D41 (2,a); UV/K14 (1,a); HaCat (1,a); SV/K14 (1,a) |

Controls: human fetus, FTF (2,a); human term placenta, 3ASubE (1,a); human skin fibroblast, Malme3 (1,a); murine fibroblast (Swiss) 3T3 (2,a); murine epidermoid carcinoma SHINOGI (1,a); murine rectal carcinoma, CMT (1,a). Cell line characteristic: (1) established; (2) finite. Culture media source: (a) authors; (b) J. Masters, Institute of Urology; (c) B. Hill, Imperial Cancer Research Fund; (d) M. Turkish, Surgical Unit, The London Hospital; (e) P. Lavender, Chemical Endocrinology Department, St Bartholomew's Hospital. Neoplastic finite cultures are initial cell cultures from tumours where lines have not yet been fully characterised. Established non-neoplastic cell lines had transformed spontaneously (HU609; HS0767; Malme-3; HaCAT) by SV/40 virus infection (SV/40; 3aSubE) and SV/40 virus followed by UV light exposure (UV/K14).

Correspondence: R.K. Iles.
Received 15 June 1989; and in revised form 15 December 1989.

*Immunological characterisation*

All samples were initially assayed using a radioimmunoassay (RIA) directed against the specific beta-subunit. The polyclonal anti-beta-hCG antibody (S424, ILS Ltd, London, UK) employed in this assay recognises both free beta-subunit and intact hormone and has been described by Norman *et al.* (1985). Material reactive in this assay is therefore referred to as 'beta'-hCG. Standards were obtained from the National Institute of Biological Standards, Potters Bar, Hertfordshire, UK (intact hCG, Third International Standard Preparation 75/537). Free beta-subunit is used as tracer (NIH preparation CR123). The lowest standard is 15 mIU $ml^{-1}$ in this assay but a detection limit of 25 mIU $ml^{-1}$ has been used. Both intact and free beta-hCG give equal displacement (100% cross-reaction); alpha subunit and LH show a 2% cross-reaction. The common alpha-subunit was estimated by the RIA of Hagen *et al.* (1976). This assay uses alpha-hCG (NIH preparation CR123) as standard and tracer. The polyclonal rabbit anti-alpha-hCG antibody was provided by Dr J.G. Pierce (UCLA School of Medicine). All of the glycoprotein hormone alpha-subunits give equal displacement. Intact hCG and LH show an 8% cross-reaction and beta-hCG less than 1%. Intact hCG was detected using a highly specific qualitative two-site immunoenzymometric assay (Tandem Icon II hCG; Hybritech Inc., San Diego, CA, USA). This employs an immobilised monoclonal antibody to the alpha-subunit as the capture antibody and a peroxidase-linked anti-beta-subunit antibody for detection. The detection limit is 25 mIU $ml^{-1}$. All assays were validated for use with tissue culture medium (Iles & Chard, 1989).

### Results

Immunoreactive hCG was detected in the culture medium of choriocarcinoma (3/3), bladder cancers (7/10), oral and genital SCC and EC (6/12), lung carcinomas (4/5), 'normal' urothelium (6/8) and 'normal' oral mucosa (3/4). Low levels of hCG were also detected in the culture media of one of eight skin keratinocyte cell lines and a control culture of fetal fibroblasts (Table II and Figure 1). No hCG was detected in cell lines from testicular germ cell tumours, epithelial ovarian carcinomas, colorectal carcinomas and breast carcinomas. Partial characterisation of the immunoreactive hCG is shown in Table II. Material produced by choriocarcinoma cell lines and the fetal fibroblast line reacted in the beta hCG, free alpha subunit and intact hCG assays. All other culture media reacted only in the beta hCG assay.

### Discussion

Ectopic production of bioactive hCG-like material by non-gestational tumours *in vivo* was reported as early as 1946 (McFadzean, 1946). Since the introduction of much more specific and sensitive immological assays directed towards the beta-subunit of the hormone many more tumours, particularly those of germ cells, have been recognised as hCG producers (Braunstein *et al.*, 1973; Javadpour *et al.*, 1978*a*, *b*). The highest incidence of ectopic expression of hCG by epithelial carcinomas is found with islet cell carcinomas (45%) followed by ovarian carcinoma (39%) (reviewed by Braunstein, 1983). The specificity of detection of very low levels of hCG in association with epithelial tumours has frequently been questioned (Adejuwon *et al.*, 1980; Braunstein, 1983). In many series the levels reported ranged between 5 and 25 mIU $ml^{-1}$ serum. Most hCG immunoassays have detection limit of between 5 and 15 mIU $ml^{-1}$. The accuracy of detection and quantification at the lower end of a standard curve is poor. For this reason Braunstein (1983) suggested that true 'ectopic' hCG expression should be when levels above 25 mIU $ml^{-1}$ are found in serum. Nevertheless, using highly sensitive assay systems immunoreactive hCG has been detected in lysates of some normal tissues

**Table II** Characterisation of 'hCG' in culture medium as determined by specific immunoassays

| *Cell culture* | *Beta-hCG ($mIU\ ml^{-1}$)* | *Alpha subunit ($ng\ ml^{-1}$)* | *Intact hCG* |
|---|---|---|---|
| Choriocarcinomas | | | |
| JAR | 2500 | 18 | + |
| BeWo | 4800 | 250 | + |
| JEG-3 | 3200 | 180 | + |
| Bladder carcinomas | | | |
| SCaBER | 2400 | <0.25 | – |
| J82 | 56 | <0.25 | – |
| RT4 | 34 | <0.25 | – |
| 5637 | 220 | <0.25 | – |
| TccSUP | 34 | <0.25 | – |
| RT112 | 220 | <0.25 | – |
| TccDES | 3600 | <0.25 | – |
| Oral and genital SCC and EC | | | |
| A431 | 110 | <0.25 | – |
| TR126 | 230 | <0.25 | – |
| SCC12B | 38 | <0.25 | – |
| TR146 | 55 | <0.25 | – |
| HN-1-P | 440 | <0.25 | – |
| CaSk1 | 40 | <0.25 | – |
| Lung carcinomas (small cell) | | | |
| Martin | 35 | <0.25 | – |
| Frei | 75 | <0.25 | – |
| Pocock | 55 | <0.25 | – |
| Highgate | 44 | <0.25 | – |
| 'Normal' urothelium | | | |
| HU609 | 1150 | <0.25 | – |
| NB110 | 42 | <0.25 | – |
| NB/JOH | 105 | <0.25 | – |
| NB/217 | 45 | <0.25 | – |
| NB/U1 | 60 | <0.25 | – |
| NB/AJ | 130 | <0.25 | – |
| NB/U2 | 70 | <0.25 | – |
| 'Normal' oral mucosa | | | |
| Gum.BL | 42 | <0.25 | – |
| OrMu A | 145 | <0.25 | |
| OrMu B | 115 | <0.25 | |
| Skin keratinocyte | | | |
| HaCAT | 32 | <0.25 | – |
| Control (Human) | | | |
| FTF | 34 | 8 | + |

**Figure 1** Incidence and levels of immunoreactive hCG secreted into the culture media by cell lines of different tumour/tissue origin. Cell lines: 1, testicular germ cell tumours; 2, ovarian carcinoma; 3, choriocarcinomas; 4, bladder carcinoma; 5, colorectal carcinomas; 6, breast carcinomas; 7, oral and genital epidermoid and squamous cell carcinomas; 8, lung carcinomas; 9, 'normal' urothelium; 10, 'normal' oral mucosa; 11, skin keratinocytes; 12, human controls; 13, murine controls.

(Yoshimoto *et al.*, 1979*a*) and also at extremely low levels (0.4–7.9 mIU $ml^{-1}$) in the serum of post-menopausal subjects (Odell & Griffin, 1987). Most *in vitro* studies of ectopic hCG

expression by non-trophoblastic/germ cell tumours have also used highly sensitive assays (i.e. Ruddon *et al.* (1979), 0.5 mIU $ml^{-1}$; Rosen *et al.* (1980), 0.02 mIU $ml^{-1}$). In the present study we have used a conservative cut-off point suggested by Braunstein (1983) (25 mIU $ml^{-1}$) for detection of hCG-like material.

Substantial qualitative variability in the hCG material produced eutopically and ectopically has been reported (Yoshimoto *et al.*, 1979*b*). There often is an imbalance of hCG subunit expression in both gestational and germ cell tumours (Gaspard *et al.*, 1980; Norman *et al.*, 1985). Furthermore, excessive production of free beta subunit appears to be a prognostic indicator, identifying patients with high risk gestational trophoblastic disease (Khazaeli *et al.*, 1989). Independent subunit expression is especially characteristic of epithelial tumours (Weintraub & Rosen, 1973; Rosen & Weintraub, 1974). However, *in vitro* studies do not always parallel *in vivo* findings: for example, testicular germ cell tumours have a high incidence of hCG expression *in vivo*, but do not express the hormone when grown in long-term tissue culture (Andrews *et al.*, 1980; Iles *et al.*, 1987). Previous studies of tumour cell lines *in vitro* have reflected this unbalanced expression of hCG subunits by neoplastic epithelia (Ruddon *et al.*, 1979; Rosen *et al.*, 1980). In a previous study we have shown that the hCG-like material secreted by 'normal' and neoplastic bladder epithelial cell lines consisted almost entirely of free beta-subunit (Iles & Chard, 1989). The partial characterisation described here strongly suggests that the hCG-like material produced by some squamous cell, small cell and epidermoid carcinomas and by 'normal' oral mucosa is also largely free beta subunit. This is in agreement with the findings of Cowley *et al.* (1985). The hCG-like material isolated from serum and urine of patients with bladder cancer has also been shown to consist mainly of free beta subunit, though smaller molecular weight forms have been noted in urine (Hattori *et al.*, 1980; Norman *et al.*, 1985; Rodenburg *et al.*, 1985).

The metabolic clearance of hCG from the circulation results in the formation of a number of products including free beta subunit, asialo free beta subunit and free beta core (Wehmann & Nisula, 1980; Blithe *et al.*, 1988). These are collectively known as urinary gonadotrophin fragments (UGF) and are readily detected in pregnancy urine and that of patients with trophoblastic disease (Kato & Braunstein, 1988; Birken *et al.*, 1988). The smaller molecular weight urinary beta core fragment is the sole measurable hCG related cancer marker in some patients with non-trophoblastic disease (Papapetrou & Nicopoulou, 1986). Indeed, beta core fragment can be detected in the urine of normal healthy men (Kardana *et al.*, 1988). This has led to the suggestion that beta core may not simply be a metabolite but may actually be synthesised by the trophoblast and a variety of neoplastic and normal tissues (Kardana *et al.*, 1988; Cole *et al.*, 1988; Cole & Birken, 1988). The 'beta' hCG RIA used in the current studies detects all these fragments but exact cross-reactivity cannot be calculated due to non-parallelism by these fragments. Further characterisation is necessary to determine whether one or all of these are present (Iles & Chard, unpublished data). Most beta-hCG positive samples were parallel on dilution. Variation on dilution encountered in some samples is possibly due to the differing affinity of our assay for epitopes recognised in complex mixtures of intact hCG, free beta, asialo free beta and beta-core. However, we have previously identified whole beta subunit as the major constituent hCG in the culture medium of 'normal' and neoplastic urothelium (Iles & Chard, 1989).

This study suggests that expression of the free beta subunit of hCG (or related UGF) is characteristic of some neoplastic and normal epithelial cells from mucosal tissues. The genetic complexity of the beta-hCG-LH gene-pseudogene cluster makes it difficult to assess whether the same gene(s) active in the placenta are also those expressed by these tissues (Iles *et al.*, 1989). It is of interest to note that the epithelial tumour groups which did not secrete hCG-like material (breast, colorectum and ovary) are generally adenocarcinomas, while the secreting carcinomas (bladder, lung, etc.) have squamous metaplastic histology. Indeed, a recent histochemical study has shown that beta-hCG expression by bladder tumours correlates with the presence of squamous cell metaplasia (Martin *et al.*, 1989). As stated *in vitro* results do not always correspond to *in vivo* findings. A recent study has correlated beta-hCG expression by colonic cancers with poor prognosis (Yamaguchi *et al.*, 1989). None of the five colorectal cancer lines studied here were found to secrete hCG-like material. Despite this, it is possible to speculate that ectopic beta-hCG expression may be a phenomenon of transitional cell/squamous cell metaplasia. The fact that primary cultures of normal urothelium and oral mucosa also produced this peptide, further indicates that this is not solely due to neoplastic events.

We are most grateful to Dr J. Masters and Dr B. Hill, and Mr M. Turkish and Mr P. Lavender for supplying us with tissue culture media from various cell lines.

### References

ADEJUWON, C.A., KOIDE, S.S., MITSUDO, S.M. & SEGAL, S.J. (1980). Apparent chorionic gonadotrophin immunoreactivity in human non-placental tissues. In *Chorionic Gonadotropin*, Segal, S.J (ed.) p. 411. Plenum: New York.

ANDREWS, P.W., BRONSON, D.L., BENHAM, F., STRICKLAND, S. & KNOWLES, B.B. (1980). A comparative study of eight cell lines derived from human testicular teratocarcinoma. *Int. J. Cancer*, **26**, 269.

BIRKEN, S., ARMSTRONG, E.G., KOLKS, M.A.G. & 5 others (1988). Structure of the human chorionic gonadotropin β-subunit fragment from pregnancy urine. *Endocrinology*, **123**, 572.

BLITHE, D.L., AKAR, A.H., WEHMANN, R.E. & NISULA, B.C. (1988). Purification of core fragment from pregnancy urine and demonstration that its carbohydrate moieties differ from those of native human chorionic gonadotropin-β. *Endocrinology*, **122**, 173.

BRAUNSTEIN, G.D., VAITUKAITIS, J.L., CARBONE, P.P. & ROSS, G.T. (1973). Ectopic production of human chorionic gonadotropin by neoplasms. *Ann. Intern. Med.*, **78**, 39.

BRAUNSTEIN, G.D. (1983). hCG expression in trophoblastic and non-trophoblastic tumours. In *Oncodevelopmental Markers. Biologic, Diagnostic and Monitoring Aspects*, Braunstein, G.D. (ed.) p 351. Academic Press: New York.

COLE, LA. & BIRKEN, S. (1988). Origin and occurrence of human chorionic gonadotropin β-subunit core fragment. *Mol. Endocrinol.*, **2**, 825.

COLE, L.A., WANG, Y., ELLIOT, M. & 4 others (1988). Urinary human chorionic gonadotropin free subunit and core fragment: a new marker of gynaecological cancers. *Cancer Res.*, **48**, 1356.

COWLEY, G., SMITH, J.A., ELLISON, M. & GUSTERSON, B. (1985). Production of human chorionic gonadotrophin by human squamous carcinoma cell lines. *Int. J. Cancer*, **35**, 575.

GASPARD, U.J., REUTER, A.M., DELVILLE, J.-L., VRINDTS-GEVAERT, Y., BAGSHAWE, K.D. & FRANCHIMONT, P. (1980). Serum concentrations of human chorionic gonadotropin and its alpha and beta subunits. 2. Trophoblastic tumours. *Clin. Endocrinol.*, **13**, 319.

HAGEN, C., GILBY, E.D., MCNEILLY, A.S., OLGAARD, K., BONDY, P.K. & REES, L.H. (1976). Comparison of circulating glycoprotein hormones and their subunits in patients with oat cell carcinoma of the lung and uraemic patients on chronic dialysis. *Acta Endocrinol.*, **83**, 26.

HATTORI, M., YOSHIMOTO, Y., MATSUKURA, S. & FUJITA, T. (1980). Qualitative and quantitative analyses of human chorionic gonadotropin and its subunits produced by malignant tumors. *Cancer*, **46**, 355.

ILES, R.K., OLIVER, R.T.D., KITAU, M., WALKER, C. & CHARD, T. (1987). *In vitro* secretion of human chorionic gonadotrophin by bladder tumour cells. *Br. J. Cancer*, **55**, 623.

ILES, R.K. & CHARD, T. (1989). Immunochemical analysis of the human chorionic gonadotrophin-like material secreted by 'normal' and neoplastic urothelial cells. *J. Mol. Endocrinol.*, **2**, 107.

ILES, R.K., CZEPULKOWSKI, B.H., YOUNG, B.D. & CHARD, T. (1989). Amplification or rearrangement of the beta-hCG-hLH gene cluster is not responsible for ectopic production of beta-hCG by bladder tumour cells. *J. Mol. Endocrinol.*, **2**, 113.

JAVADPOUR, N., MCINTIRE, K.R. & WALDMANN, T.A. (1978*a*). Immunochemical determination of human chorionic gonadotropin (hCG) and alphafetoprotein (AFP) in sera and tumours of patients with testicular cancer. *Natl Cancer Inst. Monogr.*, **49**, 209.

JAVADPOUR, N., MCINTIRE, K.R. & WALDMANN, T.A. (1978*b*). Human chorionic gonadotropin (hCG) and alpha-fetoprotein in sera and tumour cells of patients with testicular seminoma. *Cancer*, **42**, 2768.

KARDANA, A., TAYLOR, M.E., SOUTHALL, P.J., BOXER, G.M. ROWAN, A.J. & BAGSHAWE, K.D. (1988). Urinary gonadotrophin peptide-isolation and purification, and its immunohistochemical distribution in normal and neoplastic tissues. *Br. J. Cancer*, **58**, 281.

KATO, Y. & BRAUNSTEIN, G.D. (1988). Core fragment is a major form of immunoreactive urinary chorionic gonadotropin in human pregnancy. *J. Clin. Endocrinol. Metab.*, **66**, 1197.

KHAZAELI, M.B., BUCHINA, E.S., PATTILLO, R.A. SOONG, S.J. & HATCH, K.D. (1989). Radioimmunoassay of free β-subunit of human chorionic gonadotropin in diagnosis of high-risk and low-risk gestational trophoblastic disease. *Am. J. Obstet. Gynecol.*, **160**, 444.

MARTIN, J.E., JENKINS, B.J., ZUK, R.J., OLIVER, R.T.D. & BAITHUN, S.I. (1989). Human chorionic gonadotrophin expression and histological findings are predictors of response to radiotherapy in carcinoma of the bladder. *Virchows Arch. A Pathol. Anat.*, **414**, 273.

MCFADZEAN, A.J.S. (1946). Feminisation associated with carcinoma of the adrenal cortex. *Lancet*, **ii**, 940.

NORMAN, R.J., LOWINGS, C., OLIVER, T. & CHARD, T. (1985). Human chorionic gonadotrophin and its subunits - heterogeneity in serum of male patients with tumours of the genital tract. *Clin. Endocrinol.*, **23**, 25.

ODELL, W.D. & GRIFFIN, J. (1987). Pulsatile secretion of human chorionic gonadotropin in normal adults. *N. Engl. J. Med.*, **317**, 1688.

PAPAPETROU, P.D. & NICOPOULOU, S.C. (1986). The origin of a human chorionic gonadotrophin β-subunit-core fragment excreted in the urine of patients with cancer. *Acta Endocrinol.*, **112**, 415.

RODENBURG, C.J., NIEUWENHUYZEN KRUSEMAN, A.C., DEMAAKER, H.A., FIEUREN, E.J. & VAN OOSTEROM, A.T. (1985). Immunohistochemical localization and chromatographic characterization of human chorionic gonadotrophin in bladder carcinoma. *Arch. Pathol. Lab. Med.*, **109**, 1046.

ROSEN, S.W. & WEINTRAUB, B.D. (1974). Ectopic production of the isolated alpha subunit of the glycoprotein hormones. *N. Engl. J. Med.*, **290**, 1441.

ROSEN, S.W., WEINTRAUB, B.D. & AARONSON, A. (1980). Nonrandom ectopic protein production by malignant cells: direct evidence *in vitro*. *J. Clin. Endocrinol. Metab.*, **50**, 834.

RUDDON, R.W., ANDERSON, C., MEADE, K.S., ALDENDERFER, P.H. & NEUWALD, P.D. (1979). Content of gonadotropins in cultured human malignant cells and effects of sodium butyrate treatment on gonadotropin secretion by Hela cells. *Cancer Res.*, **39**, 3885.

WEHMANN, R.E. & NISULA, B.C. (1980). Characterization of a discrete degradation product of human chorionic gonadotropin β-subunit in humans. *J. Clin. Endocrinol. Metab.*, **51**, 101.

WEINTRAUB, B.D. & ROSEN, S.W. (1973). Ectopic production of the isolated beta subunit of human chorionic gonadotrophin. *J. Clin. Invest.*, **52**, 3135.

YAMAGUCHI, A., ISHIDA, T., NISHIMARA, G. & 5 others (1989). Human chorionic gonadotropin in colorectal cancer and its relationship to prognosis. *Br. J. Cancer*, **60**, 382.

YOSHIMOTO, Y., WOLFSEN, A.R., HIROSE, F. & ODELL, W.D. (1979*a*). Human chorionic gonadotrophin-like material: presence in normal human tissues. *Am. J. Obstet. Gynecol.*, **134**, 729.

YOSHIMOTO, Y., WOLFSEN, A.R. & ODELL, W.D. (1979*b*). Glycosylation. A variable in the production of hCG by cancers. *Am. J. Med.*, **67**, 414.